# Purification, Characterization and Antioxidant Activities of Enzymolysis Polysaccharide from *Grifola frondosa*

**Published:** 2017

**Authors:** Zhao Ting, Fan Yina, Mao Guanghua, Feng Weiwei, Zou Yanmin, Zou Ye, Yang Liuqing, Wu Xiangyang

**Affiliations:** a *School of Chemistry and Chemical Engineering, Jiangsu University, Zhenjiang 212013, P.R.China. *; b *School of Pharmacy, Jiangsu University, Zhenjiang 212013, P.R. China. *; c *School of Food and Biological Engineering, Jiangsu University, Zhenjiang 212013, P.R.China. *; d *School of Environment and safety, Jiangsu University, Zhenjiang 212013, P.R.China.*

**Keywords:** Polysaccharide from *Grifola frondosa*, Enzymolysis, Purification, Characterization, Antioxidant Activities

## Abstract

Our previous study revealed that the antioxidant activity of polysaccharide (coded as FGFP) extracted from *Grifola frondosa* by enzymolysis treatment was significantly superior than that (coded as GFP) extracted by boiling-water. In this study, one purified polysaccharide fractions (coded as FGFP-11) was obtained from FGFP by purified using DEAE-52 column and Sephacryl S-500HR column. Results indicated that FGFP-11 with MW of 59.82 kDa consisted of mannose, glucose and galactose with a molar ratio of 1.00:16.36:5.25. Fourier Transform Infrared Spectroscopy (FTIR spectrum) of FGFP-11 was similar with that of polysaccharide extracted by boiling-water from *Grifola frondosa*. These indicated the enzymolysis did not destroy the polysaccharide structure. NMR spectrum showed that FGFP-11 possess α-(1→6) glycosidic bond and α-(1→3) glycosidic bond configuration. The experiment of Congo red also revealed that FGFP-11 had triple helix stereo-configuration. Moreover, the antioxidant activities of FGFP-11 were improved compared with that of GFP, especially in scavenging of hydroxyl radical and diphenyl picryl hydrazinyl (DPPH) radical.

## Introduction

*Grifola frondosa*, one of the edible mushrooms, is a basidiomycete fungus which belongs to the Polyporaceae family and mainly survives in the subtropics and temperate regions. It was regarded as a famous large-scale fungi owning to its potential biological effects (1), which include antitumor (2), antioxidant activities, immunomodulatory, antihypertensive, antidiabetic and antilipemic (3). These beneficial biological activities of *Grifola frondosa* would be attributed to its active polysaccharide which generated interest for potential researchers. It has been reported that the molecular weight (Mw) of polysaccharides from *Grifola frondosa *was very large. However, the studies by Chen *et al*. (4) indicated that the lower Mw of polysaccharides from *G. frondosa* showed better anti-tumor activities. In our previous study (5), the polysaccharide (FGFP) extracted by enzymolysis treatment from *Grifola frondosa * exhibited improved scavenging of hydroxyl radicals, DPPH radicals and 2,2›-azino-bis(3-ethylbenzothiazoline-6-sulphonic acid (ABTS) radicals compared with the polysaccharide (GFP) extracted by boiling-water. In order to further investigate the structure and activities of the FGFP, this study was undertaken to isolate and purify polysaccharide fraction from *Grifola frondosa*, and to investigate its structural and antioxidant activities. It was meaningful for developing a functional antioxidant.

## Materials and methods

*Materials and reagents*

Fruit bodies of *Grifola frondosa* identified by Dr. Guanghua Mao were provided by Fang Ge Pharmaceutical Co., Ltd. (Zhejiang Province). The fruit bodies were dried at 60 °C for 24 h and then crushed or ground into powdered form to be able to sieve through a 200 size mesh. The powder was then defatted with petroleum ether at 60 °C for 15 h (6). Commercial enzymes (including cellulose, pectinase and pancreatin) employed for the study were purchased from Hemei Biology Co., Ltd (Jingning, Shandong, China). Dextrans of different molecular weights (T-10, T-40, T-70, T-500, and T-2000) were obtained from Pharmacia Co., Ltd. DEAE-52 cellulose were obtained from Whatman Co., Ltd. Sephacryl S-500 were obtained from Pharmacia Co., Ltd. 

*Extraction and purification of polysaccharides from Grifola frondosa*

The crude polysaccharides from *Grifola frondosa* were extracted using enzymolysis treatment and boiling-water method, as described by Fan *et al.* (5). Briefly, the polysaccharides were extracted three times with the boiling water (1:10, w/v) for 3 h. The extracts were filtered and concentrated using a rotary evaporator under reduced pressure. The 95% ethanol was added to the extracts with a ratio of 5 : 1 (v/v). The precipitates were collected and freeze-dried to obtain the crude polysaccharide extracts using boiling water. The polysaccharides were treated with the combined enzymes (3%, cellulose, pectinase and pancreatin with a ratio of 2 : 2 : 1). The enzymolysis extration were carried out at 50 °C for 30 min, and the temperature was then rapidly increased to 100 °C for additional 2.5 h. The extracts were submitted to the same steps that were taken in the boiling water extraction.

The crude polysaccharides extracted using boiling water and enzymolysis treatment were deproteinated with 15% trichloracetic acid at 4 °C for 4 h, respectively. The products were dialyzed against flowing water for 12 h and then deionized water for 24 h. The non-dialyzable phase was concentrated and freeze-dried to afford deproteinated polysaccharide and coded as GFP and FGFP. 200 mg of FGFP was then dissolved in distilled water and loaded onto a cellulose DEAE-52 anion - exchange chromatography column (1.6 cm × 50 cm) which was eluted with deionized water and NaCl solutions of different concentrations (0.02, 0.05, 0.10 and 0.20 mol/L) at the flow rate of 1.0 mL/min. The fractions were classified according to the carbohydrate content and quantified using the phenol-sulfuric acid method (7). The one main fraction (FGFP-1) was obtained from FGFP. Then the FGFP-1 was further purified with Sephacryl S-500HR column (1.6 cm × 50 cm) eluted with deionized water at a flow rate of 0.50 mL/min, respectively. The purified fraction (FGFP-11) presented as a single symmetrical peak.

*Homogeneity and molecular weight determination*

The homogeneity and molecular weight of the purified fractions were determined by high-performance gel-permeation chromatography (HPGPC) on LC-10ATvp chromatograph (Shimadzu, Tokyo, Japan) that was equipped with refractive index detection (RID-10A), TSK guard column PWH (φ 7.5 mm × 75 mm; Tosoh Corporation, Tokyo, Japan) and TSK-GEL G4000PW column (7.5 mm × 300 mm; Tosoh Corporation, Tokyo, Japan). The column oven was controlled by the Shimadzu Class-VP 5.0 chromatography workstation, and the temperature kept at 30 °C. 10 *μ*L sample solution was injected in each run, eluted with 0.003 mol/L sodium acetate solution at a flow rate of 0.80 mL/min. The standard curve was established with T-series dextrans of known M_W_ (T-10, T-40, T-70, T-500, T-2000 and blue dextran T-2000). Molar mass at a peak maximum (M_W_) of FGFP-11 was read from the calibration curve which was plotted as log M_W _VS. Partition coefficient *K*_av_. Partition coefficient (*K*_av_) was calculated according to the following equation: 

*K*_av_ *= (Ve –Vo)/(Vt-Vo)*

Where *Ve –* elution volume, the retention time of the peak maximum, read from elution profile; *Vo *– void volume, the retention time of blue dextran (7.54 min); *Vt – *total column volume, the retention time of glucose (14.50 min).

*Monosaccharide composition*

FGFP-11 was hydrolyzed with 2 mol/L H_2_SO_4_ at 105 °C for 8 h, respectively. The hydrolysate was neutralized with excess of BaCO_3_, centrifuged and the supernatant was lyophilized. The released monosaccharides were acetylated with pyridine-acetic acid. The acetylation products were further analyzed with GC on a Shimadzu 2010 instrument equipped with a ﬂame-ionization detector. A RTS-5 column (30 m × 0.32 mm) was used with a heating program of 130 °C (5 min) to 240 °C (5 min) at a rate of 4 °C/min. The injector and detector heater temperatures were 280 °C and 300 °C, respectively. 

The rate of carrier gas (N_2_) was 50 mL/min. The standard monosaccharides were measured using the same procedure above.

*FTIR spectrophotometer*

The FGFP-11 was ground with Potassium Bromide powder and pressed into pellets, respectively. The FTIR spectra were recorded in the region of 4000 – 500 cm^−1 ^using a Fourier transform infrared spectrophotometer (Nicolet Avatar - 370, USA)(8).

*NMR spectroscopy*

NMR spectrum was recorded using a Bruker 400 NMR spectrometer (Bruker, Rheinstetten, Germany) in D_2_O at room temperature. The FGFP-11 samples were lyophilized three times with D_2_O solution prior to the start of experiments. The chemical shift was expressed in ppm (9).

*Helix-coil transition assay*

The conformational structure of the FGFP-11 in solution was determined using characterizing Congo red-polysaccharide complexes. The transition from a triple-helical arrangement to the single-stranded conformation was examined by measuring the λ_max_ of Congo red-polysaccharide solutions at NaOH concentrations ranging from 0 to 0.50 mol/L. FGFP-11 samples (5 mg) were dissolved in 2 mL distilled water and mixed with 80 *μ*mol/L Congo red solution (2.00 mL). Drops of 1 mol/L NaOH solution was added to above mixed solution to make the final concentration of NaOH in the mixed solution being 0.00 mol/L, 0.10 mol/L, 0.15 mol/L, 0.20 mol/L, 0.25 mol/L, 0.30 mol/L, 0.35 mol/L, 0.40 mol/L, 0.45 mol/L and 0.50 mol/L respectively. UV-visible spectra of the mixture at various concentration of the NaOH were scanned with the UV-visible spectrophotometer (TU-1800, China) at 400-800 nm and the maximum absorption wavelength was recorded. In other reaction system, distilled water, instead of FGFP-11 solution, was mixed with Congo red and NaOH solution. The visible spectra were also scanned using the same method as that used for the distilled water mixed with Congo red and NaOH solution (10, 11).

*Antioxidant activities*

*DPPH radical scavenging assay*

The DPPH free radical scavenging activity of each sample was determined according to the method described by Qiao *et al*.(12) with some modifications. 2.00 mL DPPH· solution (2 × 10^-4^ mol/L in dehydrated alcohol) was added to the different concentrations of polysaccharide solution (2.00 mL). The mixture was shaken and allowed to settle for 30 min in the dark. In this assay, ascorbic acid was used as a standard antioxidant to validate the assay. The IC_50_ value (concentration providing 50 % inhibition) was graphically determined using a calibration curve in the linear range by plotting the extract concentration against the corresponding scavenging effect. The DPPH radical scavenging effect was determined using the following equation: Scavenging rate = [1 - (A_i _- A_j_) /A_0_] ×100%. 

*Hydroxyl radical scavenging assay*

The hydroxyl radical scavenging activity was evaluated according to the method of Fan *et al*.(13) with some modifications. Different concentrations (0.10 – 1.00 mg/mL) of the samples were incubated with 6 mmol/L FeSO_4_ (2.00 mL) and 3% H_2_O_2_ (2.0 mL) for 10 min at room temperature. The hydroxyl radicals were detected at 510 nm after the salicylic acid (2.00 mL) was added to the sodium phosphate buffer (150 mmol/L; pH 7.4) for 30 min at 37 °C. As a control, the sample was substituted with ascorbic acid. The calculation formula was the same as the formula used in "section 2.8.1"

*ABTS+ radical scavenging assay*

The ABTS assay was performed according to a previous method (14) with slight modifications. The ABTS^+^ radical cation (ABTS^+^) was produced by mixing the 7 mmol/L ABTS^+^ solution with the 2.5 mmol/L potassium persulfate aqueous solution and the mixture would be kept in the dark at room temperature for 12-16 h. Before the mixture was used, the ABTS^+ ^was diluted with ethanol to an absorbance of 0.70 ± 0.02 at 734 nm. Then 3.80 mL of the ABTS^+^ solution was added to 0.20 mL of various concentrations of the sample solutions. After reaction at room temperature for 6 min, the A_734_ absorbance was measured. The calculation formula was the same as the formula used in "section 2.8.1"

*Statistical analysis*

The data were presented as the mean ± standard deviation (SD) of triplicate determinations.

## Results and discussion

*Extraction and purification of polysaccharides from Grifola frondosa*

The FGFP was fractionated with DEAE-cellulose column in order to obtain one main fractions named as FGFP-1 (eluted with deionized water). The FGFP-1 were further purified with Sephacryl S-500HR column to obtain FGFP-11. The HPGFC chromatogram of the FGFP-11 indicated that the fraction was represented by a single and symmetrical peak, indicating that each purified fraction was homogeneous polysaccharide. It was also verified with HPGFC.

**Table 1:** Comparison of Antioxidant capacity (IC_50_ values) of GFP, FGGP, FGFP-11 and other antioxidant reported by references.

**Sample**	**IC**_50_ **(mg/mL)**		
	**DPPH **	**OH**	**ABTS**
GFP	1.23 ± 0.11	8.61 ±1.28	30.06 ± 3.02
FGFP	0.66 ± 0.09	0.10 ± 0.02	1.90 ± 0.15
FGFP-11	0.61 ± 0.05	0.10 ± 0.01	2.64 ± 0.33
Vitamin C	0.01 ± 0.002	0.11 ± 0.015	0.025 ± 0.003
Polysaccharide from *floral* mushroom^[18]^	1.97	2.58	＞ 5
Polysaccharide from *Armillaria mellea, * *Calocybe gambosa,* *Clitocybe odora and * *Coprinus comatus*^[19]^	3.95, 7.08, 3.56 and 7.31	/	/
Polysaccharide from* Lentinus edodes*^[20]^	7.30	/	/
Polysaccharide from * Ganoderma applanatum*,* Ganoderma lucidum and trametes versicolor*^[21]^	＜ 0.1, 0.1 and 0.23	/	/
Polysaccharide from *Entoloma lividoalbum*^[22]^	/	0.40	/
Two polysaccharide fractions form * Tricholoma matsutake *(TM-APS-1 and TM-APS-2)^[23]^	/	0.44 and 0.27	/
Three polysaccharide fractions from * Poria cocos sclerotium *(PCP-1, PCP-2 and PCP-3)^[25]^	/	/	5.39, 8.51 and 9.52

**Figure 1:**
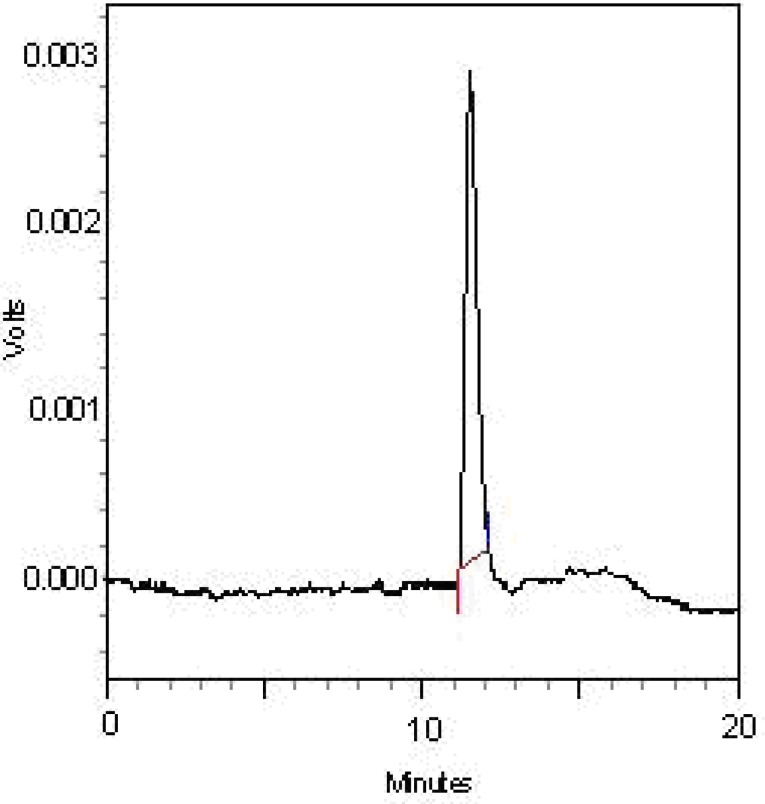
HPGPC elution profile of the FGFP-11 with refractive index detector. HPGPC was performed at 25 °C. The flow rate was 0.8 mL/min. The elution solvent was 0.003 mol/L sodium acetate solution

**Figure 2:**
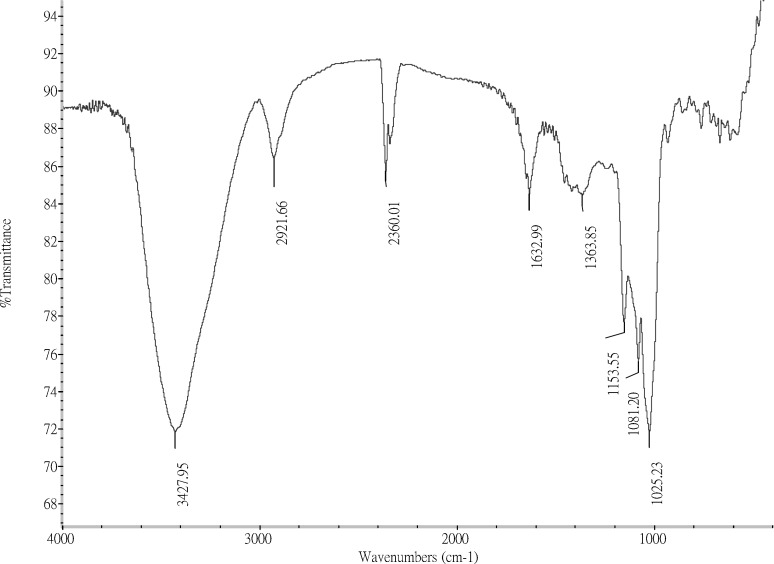
FTIR spectrum of the FGFP-11 was recorded with a NEXUS 670 FT-IR spectrophotometer between 400 and 4000 cm-1 using the Potassium Bromide-disk method

**Figure 3:**
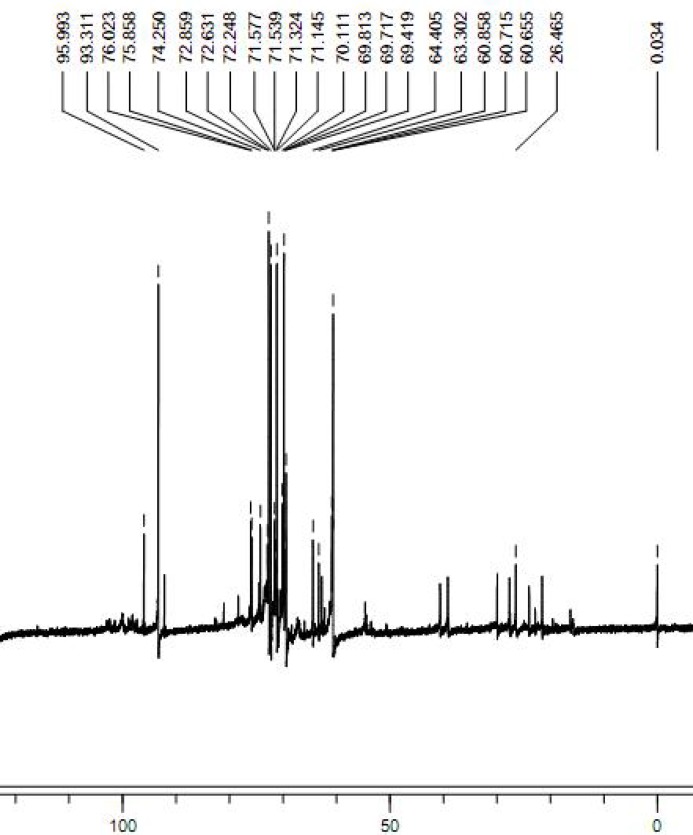
The 13C NMR spectrum of FGFP-11 was determined in a 5 mm tube using a Bruker DRX-400 spectrometer (Bruker, Rheinstetten, Germany). 13C NMR was performed at 30 °C, at 100 MHz

**Figure 4.**
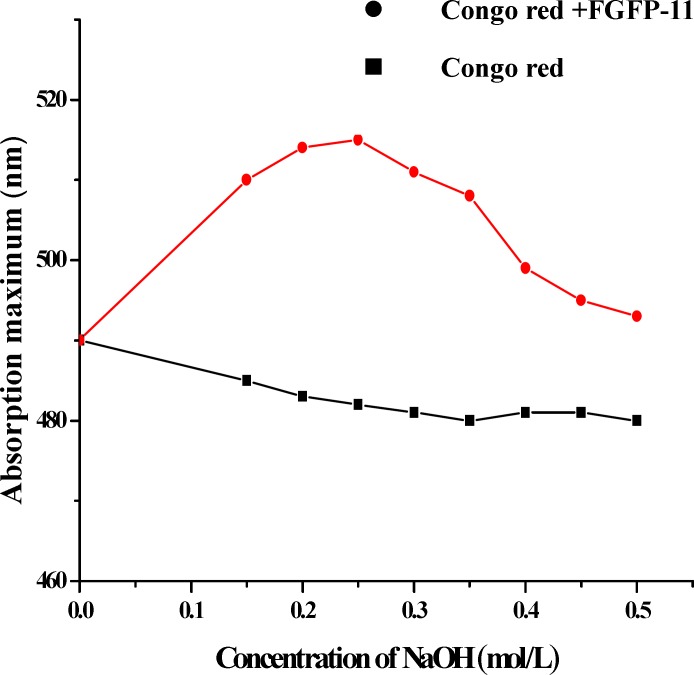
Helix-coil transition analysis of FGFP-11 according to the absorption maximum of the Congo red-polysaccharide complex at various concentrations of NaOH

**Figure 5:**
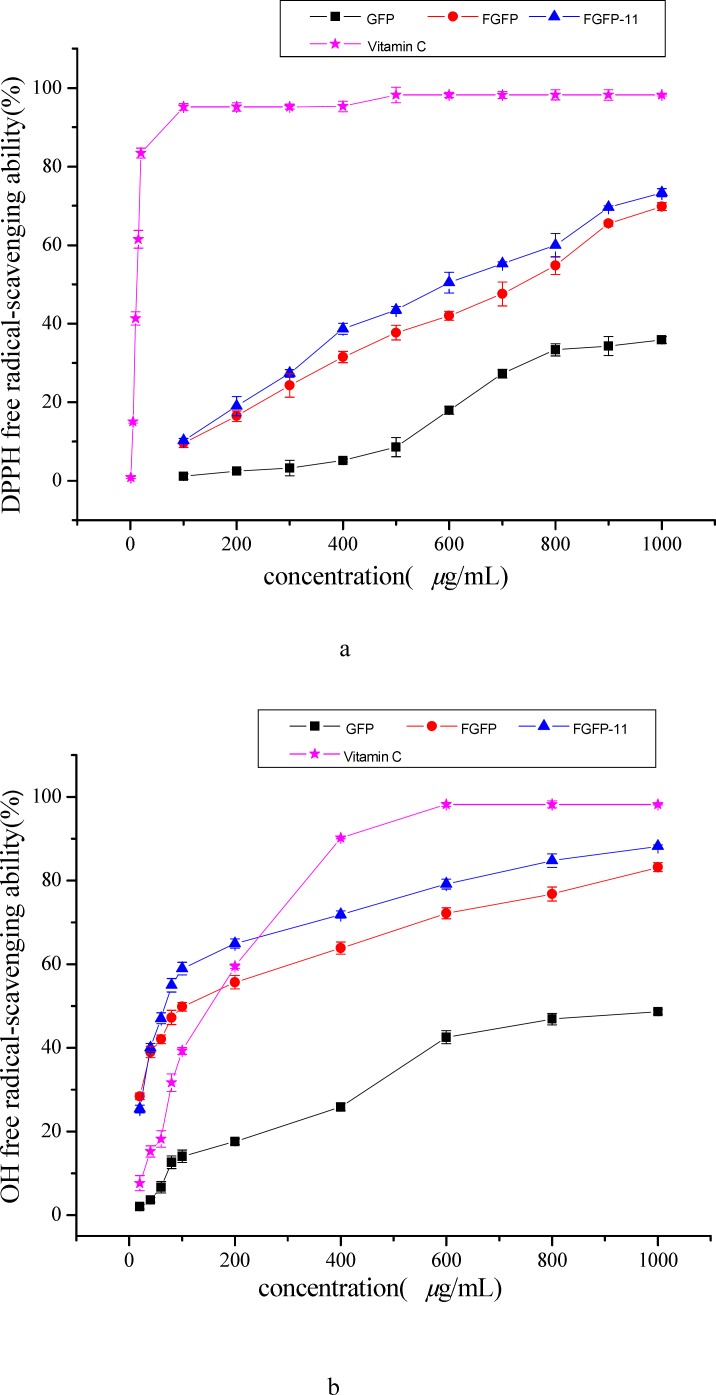
Antioxidant activities of FGFP-11 in DPPH (a), OH (b), ABTS(c) free radical-scavenging assays. Ascorbic acid was used as a positive control. Data were presented as mean ± SD (n=

*Characterization of polysaccharide fractions*

The HPGFC chromatogram of the FGFP-11 indicated that the fraction was represented by a single and symmetrical peak. The average molecular weight of FGFP-11 was 59.82 kDa. FGFP-11 was composed of mannose, glucose and galactose with molar ratio of 1.00:16.36:5.25. 

The infrared spectrum of the purified FGFP-11 is shown in Figure 2. The spectrum of FGFP-11 was similar with polysaccharide extracted by boiling-water from *Grifola frondosa*(15). They all exhibited the typical signals of polysaccharide in the range from 4000 to 500 cm^−1^. The dominance of the broad band at 3600 – 3200 cm^−1 ^could be as the results of the stretching vibration modes of OH and NH groups. The bands in the region of 2928 cm^−1 ^could be due to the CH stretching of the CH_2_ groups. The peaks between 950 cm^−1^ and 1200 cm^−1^ suggest the presence of C–O–C and C–O–H bonds. The presence of a band around 1630 cm^−1^ is characteristic of the N-H bending vibration which indicates that FGFP-11 was protein-polysaccharide complexes. 

The ^13^C NMR spectrum of the FGFP-11 was shown in Figure 3. In the ^13^C NMR spectrum of FGFP-11, most of the signals were in the region of 50.0 – 100.0 ppm. The signals of C1 revealed that the configuration of FGFP-11 was α- pyranoid sugar ring. The peaks at 95.9 ppm, 93.3 ppm and 92.1 ppm were assigned to the C1 of galactose, glucose and mannose respectively. The signals at 78.0 – 70.0 ppm were assigned to unsubstituted C2, C3, C4 and C5. The peaks at 64.0 – 60.0 ppm for FGFP-11 were attributed to unsubstituted C6. Signiﬁcant downﬁeld displacements of the signals at 82.6 ppm and 69.8 ppm for C3 and C6, as compared with their position in the spectra of corresponding nonsubstituted monomers confirm that the linkage were α-(1→6) glycosidic bond and α-(1→3) glycosidic bond(15). These data confirm the substitution pattern and further revealed the sequence of residues in the polysaccharide. The presence of polysaccharides would induce the shift in the visible absorption maximum of Congo red which is a rapid method for detecting conformational information. Generally, if the wavelength of maximum absorption of the complex with Congo red shifts to beyond 505 nm in 0.10 mol/L sodium hydroxide, it could be considered that the polysaccharide possesses a triple-helical structure in aqueous solution(10). The λ_max_ of the FGFP-11-Congo red complexes at the NaOH concentration range of 0.00 - 0.50 mol/L is shown in Figure 4. In Figure 4, it can be observed that at low concentrations (0.00 - 0.25 mol/L), the λ_max _shift to a longer wavelength, 514 nm for FGFP-11. When the concentration of NaOH was increased more than 0.25 mol/L, the λ_max _slowly dropped. This indicated that they probably adopted a highly ordered conformation, which remained stable even under strong alkaline conditions(16).

*Antioxidant activity*

*DPPH radical scavenging activity*

The model of scavenging the DPPH radical is a widely used method for evaluating the free radical-scavenging ability of various antioxidants (12, 17). Figure 5(a) shows the DPPH radical scavenging activity of the FGFP-11, FGFP and GFP. The results indicated that FGFP-11, FGFP and GFP showed dose-dependent DPPH radical scavenging activities in all concentrations. At a concentration of 1000 *μ*g/mL, the DPPH radical scavenging activity was 73.3 %-69.7 % and 35.8 % for FGFP-11, FGFP and GFP respectively. The IC_50_ values of FGFP-11 were 0.61 mg/mL, which is higher than that of Vitamin C (0.01 mg/mL), but significantly lower than that of GFP (1.23 mg/mL). It is revealed that FGFP-11 exhibited the radical scavenging activities, and was nearly twice as much as GFP in radical scavenging activity. In addition, there are some investigations reveal that polysaccharides from fungi possess the effect of scavenging the DPPH radical. Wang *et al*(18) obtain a polysaccharide (FMPS) from floral mushroom and reported that the IC_50_ value of FMPS was 1.97 mg/mL on scavenging the DPPH radical. The IC_50_ values of polysaccharides extracts from common fungi were also studied and the results showed IC_50_ values of 3.95, 7.08, 3.56, 7.31 and 7.30 mg/mL for *Armillaria mellea, Calocybe gambosa, Clitocybe odora, Coprinus comatus*(19) and *Lentinus edodes*(20), respectively. It is thus evident that the IC_50_ value of FGFP-11 was significantly lower than that above, which indicated that FGFP-11 has important value for development and utilization of antioxidant. However, there are some polysaccharides have more preferable antioxidant activity than FGFP-11, such as the polysaccharide from *Ganoderma applanatum*,* Ganoderma lucidum and Trametes versicolor*, the data of IC_50_ were 0.1, 0.1 and 0.23 mg/mL, respectively(21).

*3.3.2. Hydroxyl radical scavenging activity*

The results of hydroxyl radical scavenging activities of the two polysaccharide fractions, FGFP and GFP were shown in Figure 5(b) and compared with the frequently-used antioxidant — Vitamin C. In the concentration range of sample from 0 to 1000 *μ*g/mL, all the samples were capable of scavenging hydroxyl radical in an amount-dependent manner. The ability of FGFP-11 was strongest, followed by FGFP. GFP showed the weakest ability. At the concentration of 1000 *μ*g/mL, the scavenging effect of the FGFP-11, FGFP and GFP were 88.2 %-83.1 % and 48.6 % respectively. At the concentration of 200 *μ*g/mL, the FGFP-11 is almost three times as greater than GFP in radical scavenging activity. The IC_50_ values of the FGFP-11, FGFP and GFP were 0.10 mg /mL, 0.10 mg/mL and 8.61 mg/mL respectively. The IC_50_ values of the FGFP-11 and FGFP were close to that of Vitamin C (0.11 mg/mL) and significantly lower than that of polysaccharide from *floral* mushroom (2.58 mg/mL)(18), *Entoloma lividoalbum* (0.40 mg/mL)(22) and *Tricholoma matsutake *(0.44 and 0.27 mg/mL)(23). Therefore, these results clearly indicated that FGFP-11 has potential antioxidant ability of scavenging hydroxyl radical. 

*3.3.3. ABTS radical scavenging ability*

The ABTS assay is often used to evaluate the total antioxidant power of single compounds and complex mixtures of various plants(24). The scavenging ability of FGFP-11, FGFP and GFP on ABTS free radicals is shown in Figure 5(c). The scavenging power correlated well with increasing concentrations. FGFP-11 and GFP exhibited low radical scavenging activity at every concentration point. The effect on ABTS radical scavenging of FGFP was shown higher than those of the FGFP-11, and GFP, 50.9 % at the concentration 2000 *μ*g/mL. The IC_50_ values of the FGFP-11, FGFP and GFP were 2.64 mg/mL, 1.90 mg/mL and 30.06 mg/mL, respectively, all of them significantly higher than that of vitamin C (0.021 mg/mL), but significantly lower than that of polysaccharide from *floral* mushroom (-5 mg/mL)(18) and *Poria cocos sclerotium* (5.39, 8.51 and 9.52 mg/mL)(25). Therefore, the result showed that the FGFP-11 exhibited some ABTS radical scavenging activity.

## Conclusions

In this study two purified polysaccharides were obtained using DEAE-cellulose and Sephacryl S-500HR column from combined enzymes and boiling water. The FGFP-11 with MW of 59.82 kDa mainly consisted of mannose, glucose and galactose with a molar ratio of 1:16.36:5.25. In addition, the FTIR spectrum revealed that the FGFP-11 possess the typical signals of polysaccharide. The NMR spectrum of FGFP-11 indicated that α-(1→6) glycosidic bond, α-(1→3) glycosidic bond configuration was existed. FGFP-11 was proofed with triple helix stereo-configuration using the Congo red experiment. Moreover, the FGFP-11, and FGFP indicated improved scavenging of hydroxyl radicals, DPPH radicals and ABTS radicals when compared with GFP, and the antioxidant activities of FGFP-11 were also superior than that of other antioxidant in the reference. It is therefore recommended that FGFP-11 and FGFP could be explored as a potential novel antioxidant. However, the subtle structure, conformation and the action mechanisms of the active polysaccharide from *Grifola frondosa* require further study.
